# Application of Aluminium Flakes in Fabrication of Open-Cell Aluminium Foams by Space Holder Method

**DOI:** 10.3390/ma11081420

**Published:** 2018-08-13

**Authors:** Ioannis G. Papantoniou, Angelos P. Markopoulos, Dimitrios I. Pantelis, Dimitrios E. Manolakos

**Affiliations:** 1 Laboratory of Manufacturing Technology, School of Mechanical Engineering, National Technical University of Athens, Heroon Polytechniou 9, 15780 Athens, Greece; ipapantwniou@gmail.com (I.G.P.); manolako@central.ntua.gr (D.E.M.); 2 Shipbuilding Technology Laboratory, School of Naval Architecture and Marine Engineering, National Technical University of Athens, Heroon Polytechniou 9, 15780 Athens, Greece; pantelis@central.ntua.gr

**Keywords:** metal foam, aluminum foam, space holder method, aluminum flakes, saccharose particles

## Abstract

In the current study, a first attempt at using aluminum flakes for the manufacture of open-cell aluminum foams with the space holder method is presented. The method involves powder mixing, compaction, leaching, and sintering processes. Saccharose particles were used as space holders, and multiple parameters were investigated to optimize the manufacturing processing route in order to produce high-quality open-cell aluminum foams with a simple, economic, and environmentally friendly method. The implementation of aluminum flakes leads to foams with 80 vol.% porosity, an excellent internal open-cell porous structure, low green compaction pressures, and does not require the use of binding additives.

## 1. Introduction

Porous cellular materials have attracted strong industrial and scientific interest during the last decade. Metallic foams based on lightweight metals and specifically aluminum and its alloys, constitute promising materials for structural elements in many sectors, due to the unusual combination of properties that they offer, i.e., low weight, high energy absorption capacity, high rigidity, and high damping capacity among other properties [1,2,3,4]. These materials serve in a variety of applications, some of which are based on significant mechanical properties (mainly closed-cell foams), while others are based on rheological characteristics and transport processes, made possible by the accessibility of open pores to the ingress and flow of fluid (open-cell foams). Furthermore, the reinforcement of the metal matrix of these materials by different particles leads to special classes of metallic foams with unique and enhanced properties. Such special classes of materials are the composite and nano-composite metallic foams (CMFs) and the metal matrix syntactic foams (MMSFs) [5]. In CMFs the metal bulk matrix is incorporated with micro and nano-sized reinforcement elements to enhance the mechanical performance of the ductile metal [6,7]. Alternatively, in MMSFs, the porosity is ensured by the incorporation of ceramic micro balloons [8,9]. The micro balloons are commercially available and mainly contain various oxide ceramics [10,11].

Metal foams find many applications in areas such as the naval industry, aerospace, mechanical or chemical engineering and can be used as heat exchangers, energy or sound absorbers, filters, and implants in medicine. The use of metal foams depends on their basic characteristics such as relative density, cell structure, wall thickness, strut integrity, and cell morphology homogeneity [12,13,14]. The most common type of metallic foam is the aluminum foam, which is widely preferred due to its important mechanical and natural properties. Furthermore, two of aluminum foam’s main characteristics are recyclability and non-toxicity, which are numbered among the benefits. Low density is one more advantage of aluminum foam because of its light-weight metallic structure [15,16].

For fabrication of open-cell topology metal foams, the main manufacturing processes are: Vapor phase deposition or electro-deposition of metal into a polymer foam precursor, infiltration of a preform, use of molten metal on a replaceable precursor, and powder processing with space holder particles. During powder processing with space holder particles, porosity can be introduced by adding an additional phase (space holder particles) to the metal powder to retain spaces in the material, which can be removed afterwards by chemical or thermal (during sintering) means [17,18,19,20,21]. The shape of the space holder particles controls the pore morphology of the pore structure. Common choices for space holder particles are carbonate particles, carbamide particles, NaCl particles, expanded polystyrene granules, magnesium particles, polystyrene spheres, and, more recently, the saccharose crystals [22,23,24,25]. During the space holding method, the space-holder particles are mixed with metallic materials in powder form. Typically, the metal particles are smaller than the space-holder particles, and a small amount of an additional agent is usually needed to achieve a better mixture and binding of powders [26,27,28,29].

In the current study, a first attempt at using flake powders for the manufacture of open-cell metallic foams with the space holder method is presented. The use of aluminum flakes constitutes a novel choice of metallic material for manufacturing open-cell aluminum foams using powder processing with space holder particles. Saccharose crystals, known as crystalline raw cane sugar, were used as space-holder particles in order to form pores under a controlled processing route. The main goal of this study was to produce aluminum foams with a simple, economical, and environmentally friendly method. The main parameters, which were examined in depth for the optimization of the process parameters, and which affect the final porosity, e.g., the compaction pressure and the sintering temperature, have been set with the intention to find a correlation between aluminum foam microstructure evolution, texture modification, and the mechanical properties.

## 2. Materials and Methods

**Figure 1:**
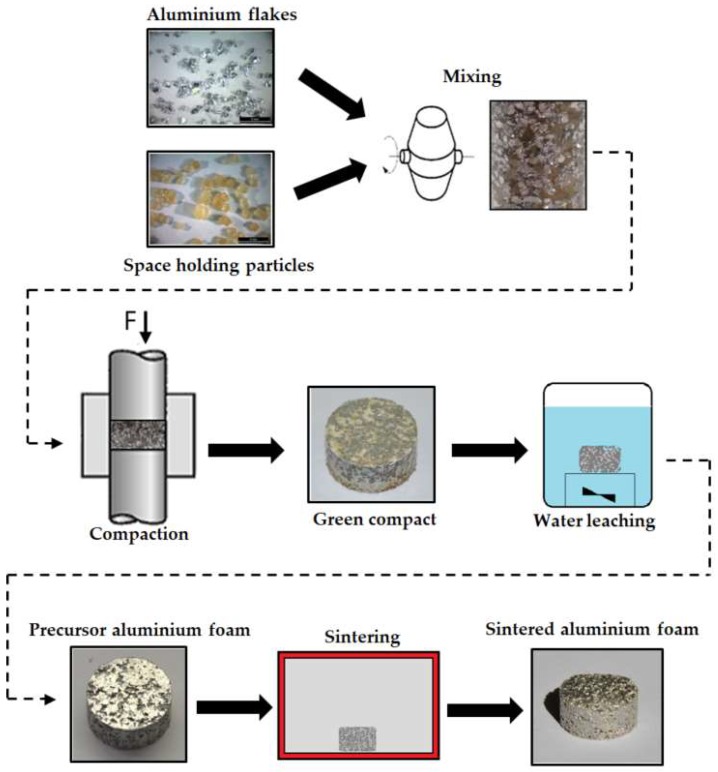
Scheme of the aluminum foam manufacturing procedure.

**Figure 2:**
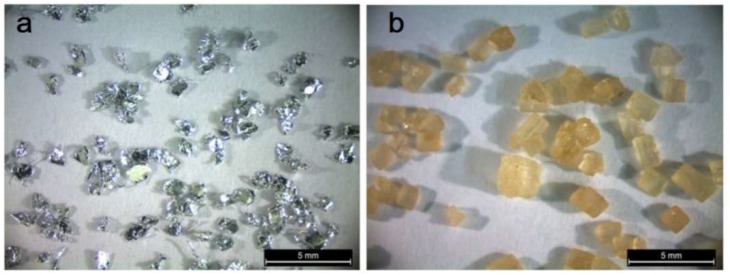
Materials used in the manufacturing process of the open-cell aluminum foam: (**a**) Aluminum flakes, (**b**) saccharose particles.

Porous aluminum specimens were produced using a space-holder, dissolution, and sintering process, as shown in Figure 1. Transmet Corporation 99.99% high purity aluminum flakes were used instead of spherical aluminum powders, see Figure 2a. The aluminum flakes presented irregular geometry with an equivalent diameter ranging from 0.45 to 1.45 mm and a mean thickness ranging from 0.016 to 0.021 mm. Saccharose crystals were used as space holding particles in order to create the intended pore size, which is dependent on the mean equivalent diameter of the crystals. The crystalline raw cane sugar particles approach cuboidal shape with a nominal mean size of 1.42 mm, as shown in Figure 2b.

The aluminum flakes and the space holding particles were mixed with a combined volume fraction of 60% and 40%, respectively. For mixing the particles, a laboratory powder mixer was used for 2 min at low speed, in order to retain the morphology of the aluminum flakes as much as possible. The mixed materials were put into a cylindrical stainless steel die, followed by a cold compaction of the mixture with the use of a mechanical press. The final green compact sample had a cylindrical geometry with a diameter of 25 mm and a height of 15 mm. After the uniaxial pressing, the compacts were placed into distilled water for the removal of the crystalline saccharose particles. At the next stage of the manufacturing procedure, the sintering process took place with the aid of an electric inductive oven, in order to strengthen the Al-matrix network of the green precursor foam, see Figure 1.

The sintered aluminum foams and the precursor foam (the non-sintered specimens) were subjected to compression experiments in order to investigate the mechanical properties of aluminum foams obtained at different sintering temperatures. The aluminum foam that illustrated better mechanical properties during the compressive experiments went through metallurgical inspection using Scanning Electron Microscopy (SEM) and X-ray Diffraction (XRD).

## 3. Results

### 3.1. Compaction

**Figure 3:**
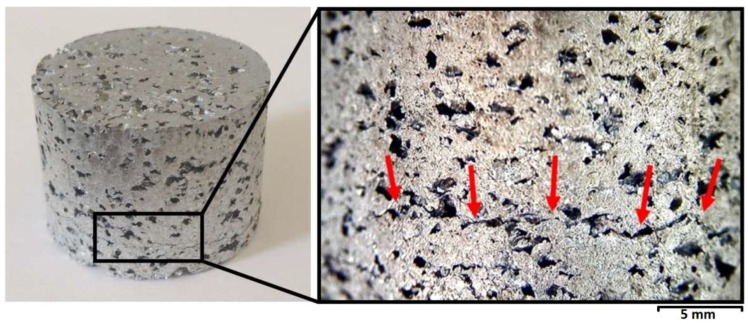
Sample set 4. Red arrows indicate a major crack caused by compaction pressures of 250 MPa.

A series of compaction experiments were carried out by varying the pressure from 100 to 250 MPa, namely 100, 150, 200, and 250 MPa, in order to find the optimum compaction pressure, see Table 1. Sample sets 1 and 2 with compaction pressures 100 and 150 MPa respectively, presented unstable bonding of the flakes which led to surface wall delamination during the dissolution stage. Sample set 4, with a compaction pressure of 250 MPa, presented no wall delamination. However, during the extraction from the die, the cylindrical samples presented cracks at mid-height, perpendicular to the pressing direction, resulting in unsteadiness of the precursor foam structures, as shown in Figure 3. The optimum green compact was produced at relatively low compaction pressures (200 MPa), and the excellent mixture of materials was easily achieved with no additional binder to assist the mixing. The applied mixing procedure at the optimum compaction pressure (Sample sets 3, 5, 6, 7, 8 and 9) produced a uniform space-holder-particle distribution, without any deterioration.

### 3.2. Dissolution

During the dissolution process, the saccharose particles were easily dissolved in water, leaving an open-cell aluminum foam green density precursor. For the space-holding particles dissolution, a magnetic stirrer with a heating plate was used at 100 rpm and 60 °C for two hours. During the dissolution, the distilled water was frequently refreshed with preheated (at 60 °C) distilled water. After the dissolution process, the precursor foams were left in a dryer for one hour in order to remove all the remaining distilled water. After the one-hour drying stage, the weight of the samples was measured using an Explorer Pro EP613C electronic balance, with an accuracy of 0.001 g, in order to ascertain if any saccharose particles remained inside the porous structure. The measurements illustrated that after the whole dissolution-drying procedure, no saccharose particles remained in the aluminum foam matrix. Finally, the green compact precursors showed high stability (absence of any surface wall delamination) after the dissolution process. This stability was enough to keep mechanical integration of the precursor metal foam during drying and placing into the furnace for sintering.

### 3.3. Sintering

**Figure 4:**
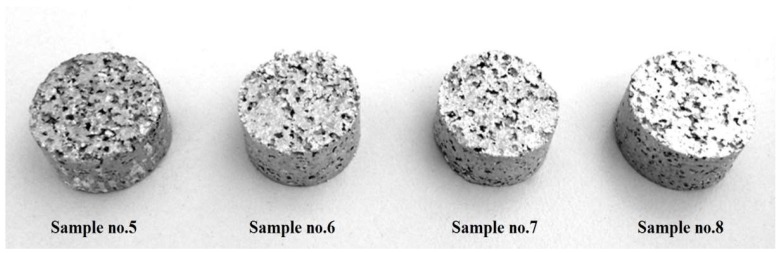
Aluminum foam specimens manufactured with different sintering temperatures.

To achieve the optimum sintering temperature, five discrete sintering temperatures were studied; 620 °C, 660 °C, 700 °C, 740 °C, and 780 °C. This range of temperatures was chosen according to literature and in order to examine the effects of undue temperatures on the sintering stage. During the sintering process, each compacted sample was firstly heated slowly at speed of 10 °C/min until the according temperatures were reached. Then, the temperature was kept stable for 3 h and finally, the samples were cooled down to room temperature with a cooling speed of 3 °C/min. Precursor foams sintered at 780 °C collapsed in the oven. The aluminum foam specimens produced at different sintering temperatures can be seen in Figure 4.

### 3.4. Evaluation of Specimens after Sintering

The theoretical porosity of the initial foam is equivalent to the volume fraction of saccharose particles in the precursor foam, but it is experimentally different from the actual porosity. This is attributed to the development of the microporous structure during compaction and the shrinkage effect during sintering. The actual porosity *P_f_* was calculated from the following equation, where the calculated density of the final Al-foam was used, dividing the mass by the volume $\left(\rho_{f}=m_{f}/V_{f}\right)$ (*V_f_* is the volume of the final foam after the sintering process) and the density of aluminum $\left(\rho_{Al}=2.7\mathrm{gr}/cm^{3}\right)$, Equation (1):

(1) $$P_{f}=1-\frac{\rho_{f}}{\rho_{Al}}$$

**Figure 5:**
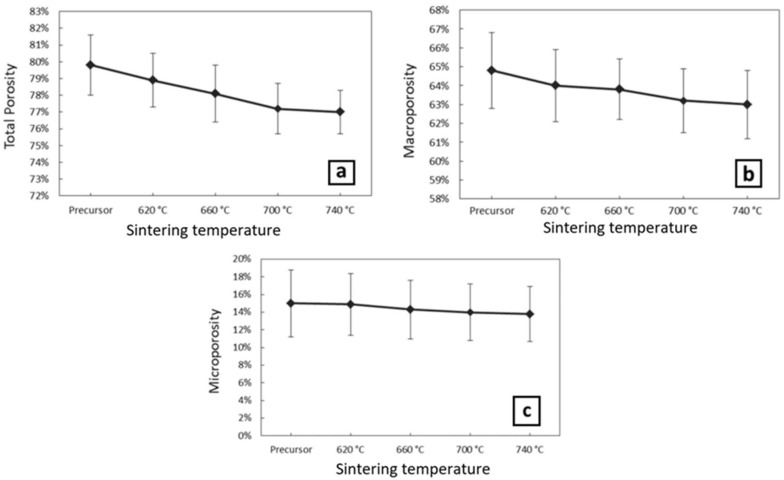
(**a**) Total porosity measured using Equation (1), (**b**) macroporosity measured using theopen source scientific image processing program, ImageJ, (**c**) microporosity calculated by subtracting the macroporosity from the total porosity.

**Figure 6:**
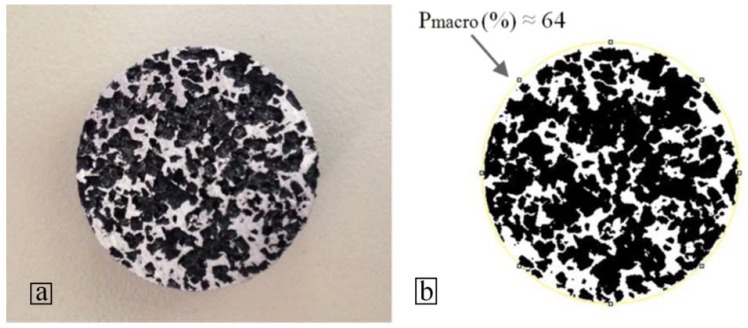
(**a**) Macrograph of a processed aluminum foam cross-section (at mid-height), (**b**) binary image of the porous structure (obtained by ImageJ).

For each sintering temperature, one set of three aluminum foams was created in order to ensure the reproducibility of the results. In all the samples the actual porosity was measured between 77–80%, as shown in Figure 5a, by using Equation (1). The actual total porosity includes the macroporosity derived from the space holder particles and the microporosity, which is caused by the micro-porous bonding structure. Therefore, in order to measure the macroporosity, the surfaces of the samples were firstly processed by Electro Discharge Machining to visualize the interior structure without introducing any smearing effects on the surface. The processed specimens were examined macroscopically with the use of an optical stereoscope (Leica MZ6). Then, by using open-source image processing software, ImageJ, binary images of the porous structure were created, and the mean macroporosity was calculated, see Figure 6. In each sample, three cross-sections were used to calculate the average value of the macroporosity. For all samples, the average macroporosity was evaluated as 63–65%, as shown in Figure 5b. Microporosity was calculated as 13–15%, as shown in Figure 5c, by subtracting the macroporosity from the total porosity. The porosity measurements diagrams indicated that there is a minor reduction of the total porosity, the macroporosity, and the microporosity with an increase of the sintering temperature.

### 3.5. Mechanical Properties of Foams

During the sintering process, the mechanical integrity of the sinter increases because high-temperature diffusion leads to bonding of the aluminum particles. After sintering, compression tests were performed in order to investigate the mechanical properties of the aluminum foams obtained at different sintering temperatures. The tests were conducted using an Instron universal tension machine at a constant crosshead speed of 5 mm/min. The samples were compressed at up to 70% strain. The machine’s axis was parallel to the direction of the compression axis and the samples were placed on the steady press base. A preload was applied to the samples until the gaps between the sample and jaws disappeared. The compressive stress was defined from Equation (2):

(2) $$\sigma =\frac{F}{\left(A\times P_{f}\right)}$$

where *F* is the compressive load, *A* is the circular base area of the cylindrical sample, and *P_f_* is the porosity of aluminum foam after the sintering process. The average value of stress in the stress-strain curve from the yield point up to the onset of densification was assigned to the plateau stress. Finally, for the calculation of the specific absorbed energy, i.e., strain energy per gram, the area under the stress-strain curve was divided to confirm the energy absorption efficiency of each sample.

Compressive experiments were performed on the precursor aluminum foam and the aluminum foams with different sintering temperatures in order to identify the effect of the sintering temperature on the mechanical properties of the foam, see Table 2. For each sintering temperature (and the precursor with no sintering process) a set of three samples was manufactured in order to study the reproducibility of the results.

**Figure 7:**
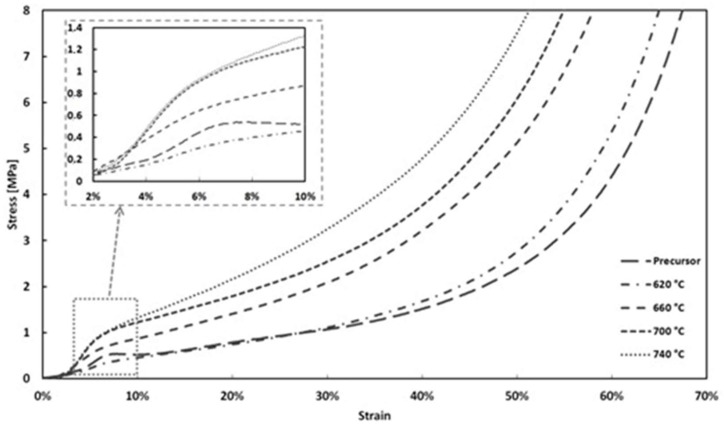
Stress–strain curves for aluminum foams in various sintering temperatures.

Figure 7 depicts the compressive stress-strain response of the Al-foam precursor and for Al-foams after the sintering process at temperatures of 620 °C, 660 °C, 700 °C, and 740 °C. The curves are characterized by the typical initial elastic response, followed by a deformation plateau with a positive slope and finally a transition to densification. Also, in the plateau region of stress-strain curves, the absence of any stress oscillations is to be noted. The non-fluctuated stress-strain curves imply a ductile deformation in the plastic region. Due to the morphology of the flakes, only a minor material separation was observed, due to a non-uniform deformation of the structure. The stress at the macroscopic yield point and the subsequent plateau stress at a certain strain, increase with increasing sintering temperature of the foam. Furthermore, one of the characteristics of the compressive stress-strain curves is a very smooth plateau region, as a result of the homogeneously distributed pores and their narrow size range. The foam with no sintering process also has a significant high plateau stress, something that was expected due to the angular morphology of the flakes and the propagation of an entanglement process between them during pressing. In order to investigate the elastic region more precisely, the elastic region was magnified from the stress-strain curves. Stress variations in the elastic region were found to be nonlinear, which can be explained by the imperfections and defects during the compaction procedure.

The compressive stress-strain curves of compressed aluminum foams illustrate the mechanical behavior, namely, the mechanical strength corresponding to the energy absorption for each of the foams. The energy absorption capacity of foams *W*, up to a strain *e*_0_, can be evaluated by integrating the area under the stress-strain curve and is calculated from Equation (3) [1,2]:

(3) $$W=\int_{0}^{e_{0}}\sigma (e)\mathrm{de}$$

In general, the energy absorption capacity increases linearly with strain until the densification strain is reached. Note that the energy dissipation in the compression behavior mainly results from two different phenomena, the mechanism of cell wall structure collapse and the evolution of friction between cell walls when they are in contact.

**Figure 8:**
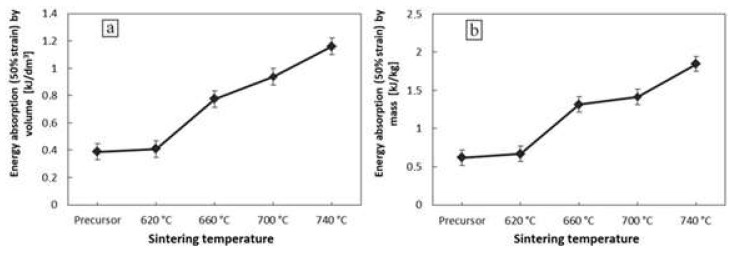
Energy absorption for 50% strain by volume (**a**) and by mass (**b**) for Al-foams with various sintering temperatures.

The variation of energy absorption by volume and mass for 50% strain in aluminum foams with various sintering temperatures is presented in Figure 8. The energy absorption is an important parameter affecting the utility of the material as an energy absorbing capacitor. The sintering temperature affects the magnitude of the compressive plateau stress. Thus, the foams sintered at higher temperatures exhibit a greater energy absorption capacity due to a higher compressive strength. Moreover, the aluminum foam introduces strong mechanical properties due to the morphology of the flakes even without the effect of the sintering process.

The efficiency of energy absorption is the effect of an ideal load-bearing porous material with perfect plastic behavior and is calculated by the equivalent area of rectangular shape, which is defined by the maximum stress (*σ_d_*) and strain (*e_d_*). Studying the energy absorption of aluminum foams, the efficiency of energy absorption, *η*, is calculated by dividing the area under the stress-strain curve (from zero point to *e_d_* point), *A_real_*, by the area that corresponds to ideal energy absorption, *A_ideal_*, Equation (4) [1,2]:

(4) $$\eta =\frac{A_{real}}{A_{ideal}}=\frac{\int_{0}^{e_{d}}\sigma (e)de}{\sigma_{d}\cdot e_{d}}$$

**Figure 9:**
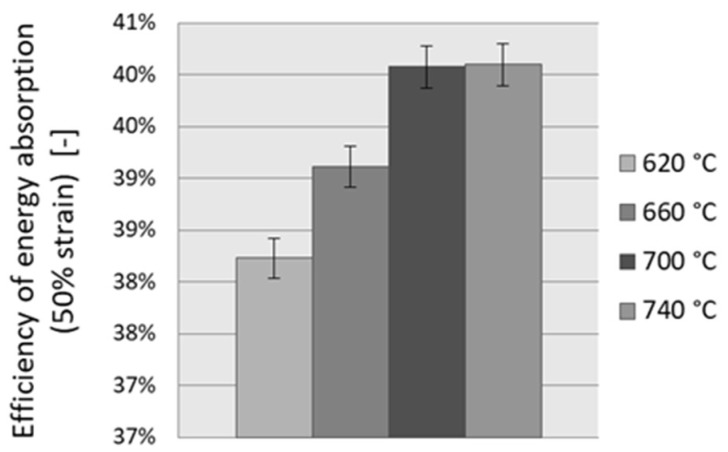
Efficiency of energy absorption for 50% strain for Al-foams with various sintering temperatures.

The efficiency of energy absorption for 50% strain is shown in Figure 9 and Table 2. The *η* value is found to increase with an increasing sintering temperature.

Conclusively, the mechanical properties, namely stress at the macroscopic yield point, plateau stress, energy absorption, and efficiency of energy absorption, are improved with the increase of the foam sintering temperature during the sintering stage. This is mainly attributed to the rupture of the oxide film of the aluminum flakes due to liquid-state sintering that led to sufficient fluidity of molten aluminum through the disrupted surfaces between the aluminum flakes and consequently to the better bonding of the flakes.

### 3.6. Microstructural Evaluation

**Figure 10:**
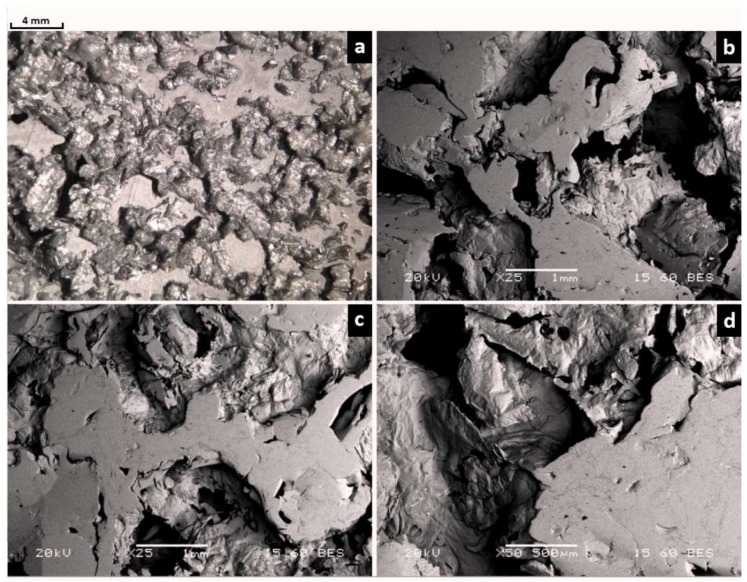
Microstructure of the aluminum foam samples sintered at 740 °C: (**a**) Optical stereoscopy image illustrating the irregular porous morphology, (**b**–**d**) scanning electron images of the interior structure of the cell wall and morphology.

The microstructure of metallic foam after sintering plays an important role in the characteristics of the final foam. The cell shape, cell size, and cell type are among the most important features that should be considered more carefully. In this paper, scanning electron microscopy was used to observe the pore morphology (irregular and angular), pore size, and pore shape due to its high-quality imaging. The microstructural characterization was performed for specimens sintered at 740 °C due to the improved mechanical properties obtained from the conducted compressive experiments. Specimens for microstructural evaluation were cut from sintered samples through Electro Discharge Machining, which was carried out parallel to the pressing direction. Subsequently, specimens were cold mounted in a high viscosity resin and subjected to a metallographic preparation, including mechanical grinding followed by polishing with diamond suspensions. The high viscosity resin was chosen in order to keep the interior pores free from resin, for better microstructural observations. For the scanning electron microscopy (SEM) examination, a Philips XL30 device at 20 kV was used; all samples were subjected to standardized metallographic mounting and polishing techniques. Figure 10a–d illustrates the typical microstructure of the cell wall of the aluminum foam samples sintered at 740 °C. The porous structure, due to the addition of space-holders, is open and interconnected. The windows are derived from the dissolution process of saccharose particles. The cell walls are not fully dense but contain some interstices that probably originated from the compaction stage and are retained after sintering (microporosity). Moreover, this process contributed to a non-uniform orientation and distribution of the pores. However, during sintering, some shrinkage occurs, which modifies the expected pore size. It is assumed that shrinkage occurs homogeneously.

**Figure 11:**
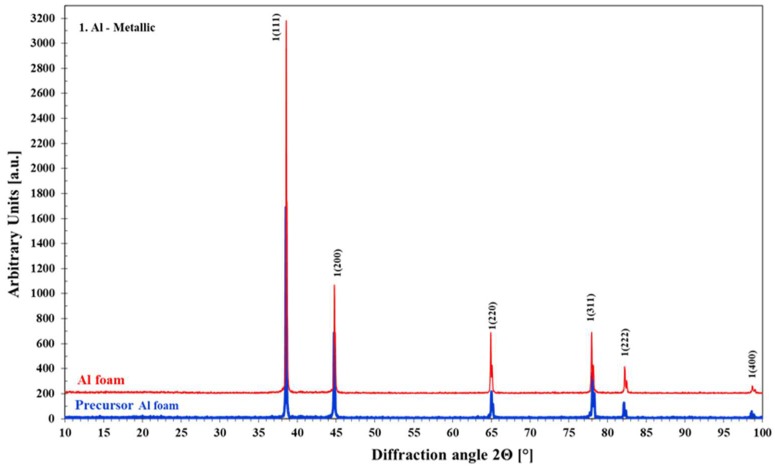
X-ray diffraction pattern for the precursor specimen and Al-foam specimen sintered at 740 °C.

Finally, phase analysis through the X-ray diffraction technique (XRD) was performed, using a Siemens D 5000 diffractometer that was equipped with a copper target. The resulting radiation was monochromated and had a wavelength of 1.54056 Å. The XRD analysis was first used to detect the existing phases and the macrotexture evolution in the metal foam before and after sintering at 740 °C. Secondly, to confirm that the saccharose particles were totally removed during the dissolution stage, as shown in Figure 11. XRD measurements revealed that with the evolution of the sintering process, a significant increment in the crystallinity factor takes place, as the diffraction peaks became sharper without any broadened feature. Finally, the XRD analysis did not reveal amorphous organic substances ascribed to the presence of raw cane sugar.

## 4. Discussion

Flake morphology powders make up a novel form of material for open-cell metal foam production using the powder processing method with space holder particles. The results illustrated many advantages corresponding to the regular spherical powders used by relevant studies [30,31,32]. More specifically, due to the morphology of the flakes, the optimum green compacted precursor was produced at relatively low compaction pressures (200 MPa), and the excellent mixture of materials was easily achieved with no additional binder needed to assist the mixing and the bonding of the green precursor. Owing to that, there was no need for sintering the precursors together with the mold. The applied mixing procedure at the optimum compaction pressure produced a uniform space-holder-particle distribution, without deterioration and any reduction in their size due to uniaxial pressure, and with a well-connected open-pore structure.

The use of flake powders in the space folding method ensures structural integrity and has the potential for many applications in the medical industry for fabricating implants with a low cost, feasible, and reliable method.

## 5. Conclusions

From the above-presented analysis, the following concluding remarks can be drawn:

- The novel use of aluminum flakes as the main material of this process allows the production of foams with an excellent internal open-cell porous structure at low compaction pressures and with no need for any binding additive.
- The saccharose crystals as a space holder can be easily removed, leaving a porous integral structure. The irregular shape and inhomogeneous morphology of the pore structure are attributed to the space holder mean size, geometry, and volume fraction.
- The mechanical properties, i.e., stress at the macroscopic yield point, plateau stress, energy absorption, and the efficiency of energy absorption, can be easily modified/improved by variation of the foam sintering temperature.
- The green compact with no sintering process presented relatively high mechanical properties due to the complex mechanical bonding of the flakes at the green density.
- The procedure introduced high reproducibility of the results with a simple, low cost, and environmentally friendly method.

## Figures and Tables

**Table 1:** Table of compaction experiments.

Samples Set (Three Samples Per Set)	Compaction Pressure [MPa]	Precursor Stability
1	100	Unstable (wall delamination)
2	150	Unstable (minor wall delamination)
3	200	Stable
4	250	Unstable (cracks)

**Table 2:** Investigated parameters in experimental scale-up processing.

Samples Set (Three Samples per Set)	Compaction Pressure [mpa]	Sintering Temperature [°C]	Energy Absorption (50% Strain) by Volume [kj/dm^3^]	Energy Absorption (50% Strain) by Mass [kj/kg]	Porosity [%]
3	200	No sintering	0.39	0.62	79.8 ± 1.8%
5	200	620	0.41	0.67	78.9 ± 1.6%
6	200	660	0.78	1.32	78.1 ± 1.7%
7	200	700	0.94	1.41	77.2 ± 1.5%
8	200	740	1.16	1.85	76.8 ± 1.3%
9	200	780	Precursor foams sintered at 780 °C collapsed in the oven		
